# Understanding the Reasons behind Anticipated Regret for Missing Regular Physical Activity

**DOI:** 10.3389/fpsyg.2016.00700

**Published:** 2016-05-10

**Authors:** Ryan E. Rhodes, Chetan D. Mistry

**Affiliations:** Behavioural Medicine Laboratory, Faculty of Education, University of Victoria, VictoriaBC, Canada

**Keywords:** anticipated affective reactions, theory of planned behavior, exercise, affective judgments, thought listing

## Abstract

Anticipated affective reactions to missing physical activity (PA), often labeled anticipated regret, has reliable evidence as a predictor of PA intention and behavior independent of other standard social cognitive constructs. Despite this evidence, the sources of regret are understudied and may come from many different reasons. The purpose of this study was to theme the reasons for why people responded to anticipated regret over missing regular PA for 2 weeks. Participants were a random sample of 120 university students who were primed on the public health definition of PA, completed measures of regret, and were asked to list their reasons for regret. Ninety-five percent of participants expressed that they would regret not being active and gave a total of 357 reasons. The dominant theme (*n* = 247; 69%) was a missed opportunity to obtain the benefits of PA, followed by shame/guilt for not being able to follow-through with one’s goals or self-categorized role (*n* = 99; 28%) with a final theme of perceived pressure from others (*n* = 11; 3%). From a practical perspective, the diversity of these reasons suggest that more clarity on the source of regret should be present in assessment, while building from both attitude and identity theories may help understand how regret motivates PA in future intervention.

## Introduction

The health benefits of regular physical activity (PA) are extensive and include reducing the risks of acquiring over 25 chronic conditions (Warburton et al., 2007). Despite this irrefutable evidence, few people engage in enough PA to reap even minimal health protective gain (Troiano et al., 2008; Colley et al., 2011). Clearly, PA promotion is of paramount concern to public health.

The use of sound theoretical approaches to understanding PA has been advocated in order to provide organized frameworks for targeted intervention (Rhodes and Nigg, 2011). Much of this theoretical research has applied social cognitive approaches that emphasize the considerable rational benefits of performing PA and enhancing control over performance (Fishbein et al., 2001). One aspect that has been under-developed in past social cognitive approaches within the PA domain is the potential role of affective processes (Rhodes et al., 2009b; Ekkekakis et al., 2013; Williams and Evans, 2014; Rhodes and Kates, 2015).

Affective expectations of enjoyment are reliable correlates of PA that add additional variance to our understanding of the behavior (Rhodes et al., 2009b; Teixeira et al., 2012). Among these sets of expectations, however, there is also evidence that anticipated affective experience from performing the behavior, may be different from the anticipated affective reaction to not performing the behavior (Conner et al., 2015). These anticipated affective reactions to abstention are typically labeled anticipated regret (Abraham and Sheeran, 2003). Our literature review of anticipated regret and PA identified seven studies that have applied the construct (Conner and Abraham, 2001; Abraham and Sheeran, 2003, 2004; Jackson et al., 2003; Sheeran and Abraham, 2003; Sandberg and Conner, 2011; Wang, 2011). Of the two experimental studies (Abraham and Sheeran, 2004; Sandberg and Conner, 2011), both showed that modifications to anticipated regret increased PA, albeit modestly. Furthermore, four of the five studies to evaluate the predictive capability of anticipated regret, showed it had a contribution to explaining intention independent of social cognitive factors in the theory of planned behavior (Conner and Abraham, 2001; Abraham and Sheeran, 2004; Sandberg and Conner, 2011; Wang, 2011). Thus, the construct has fairly reliable evidence in the PA domain as a predictor of PA.

Conner et al. (2015) propose the theoretical process for how anticipated regret affects PA is via self-conscious emotions (e.g., guilt; Giner-Sorolla, 2001). These negative emotions are anticipated pre-emptively and serve as motivation to avoid the outcome (Simonson, 1992). Interestingly, this also reflects the self-regulating processes for behavioral performance in identity theory (Stryker and Burke, 2000). Identities are considered components of a self-concept, organized by standards on how one views themselves in a given role (Burke, 2006). These standards act as comparators to actual behavior, and are activated in relevant situations where identities are either aligned or mismatched with one’s behavior. Discrepancies challenge an identity and provide negative affect that serves to motivate identity consistent behavioral actions (Stets and Burke, 2000; Price Tangney, 2003), a premise with support in the PA domain (Flora et al., 2012). Thus, prompting anticipated regret for missing PA may serve as activation of ones identity for health and well-being, or goal-driven achievement. Currently, no research has examined the reasons behind responses to PA anticipated regret, so the mechanisms remain unexamined.

While the suggested mechanisms for anticipated regret by Conner et al. (2015) seem sensible, there may also be other reasons less tied to self-conscious emotions or personal standards. For example, regret may be from mere expectations of missed opportunities. If one values the outcome of a particular behavior, failure to perform the behavior may be regretted even if there is no personal shame or guilt involved. In this case, anticipated regret may be a better measure of anticipated value of the behavior and add conceptually to the outcome expectation or attitude concept (Fishbein et al., 2001). Another possibility for regret may align more with feelings of external guilt associated with not performing the PA. This notion is commensurate with introjected regulation in self-determination theory (Deci and Ryan, 2000), where one feels a sense of obligatory duty to perform the behavior to appease others but also seems aligned with subjective norm and the perceived pressure one may feel from outside social forces (Ajzen, 1991). Given these are all plausible alternatives to the reasons for experiencing anticipated regret, a formal examination would be helpful to better articulate the construct and its antecedents.

Therefore, the purpose of this study was to theme reasons for why people respond to moderate and vigorous intensity PA anticipated regret using thought listing procedures. We also sought to explore whether participants who were regularly active had different responses from those who were active. Based on the rationale and theorizing from Conner et al. (2015), we hypothesized that most people would anticipate regretting PA due to self-conscious reasons or personal standards. Nevertheless, we hypothesized that some people may regret PA as a missed opportunity to improve health and well-being or from external pressures.

## Materials and Methods

### Participants and Procedures

Participants were students enrolled in the 2014 winter term at the University of Victoria, Canada. A list of all 100- and 200-level classes at the university were collected (*k* = 667). Classes were stratified by year and 10 different Faculties and assigned a random number between 0 and 1. The list was sorted by the random number in descending order. Within each year and Faculty, two classes with random numbers closest to 1 were selected to be contacted. Nineteen 100- and 200-level classes were randomly selected to be contacted through the university email system. The content of the email asked potential participants to complete a short survey on beliefs and motives for PA. The link for the survey was provided at the end of the email.

After informed consent, participants were asked to complete a measure of PA. On the next page of the survey, we then defined PA to create a context for subsequent questions. PA was defined as aerobic activities at least 150 min per week during free time that was in the moderate intensity (brisk pace) or higher range. Regular brisk walking was provided as an example of how one could achieve this level of PA. We also included resistance training, defined as any work with weights (squats, deadlift, bench press, curls, etc.) and other strength exercises (push-ups, pull-ups, and sit-ups) done during free time (i.e., not occupation, school, or housework). Participation in resistance training was defined as at least two bouts per week to align with Canadian public health guidelines (Tremblay et al., 2011). Following this definition of PA participants completed the anticipated regret question and thought listing procedure.

### Measures

#### Anticipated Regret

To elicit the reasons behind anticipated regret, participants were asked to consider the definition of PA noted above and then asked if they would feel regret if they did not engage in this amount of activity over the next 2 weeks. The participants were then directed to a forced dichotomy answer of a yes or no response. The phrase used in this question was identical to prior items used to asses anticipated regret in the PA domain (Abraham and Sheeran, 2003, 2004; Sheeran and Abraham, 2003), yet the forced dichotomization was created in this study to help elicit reasons for participant choices.

The thought listing measure included the statement “considering your answer on the last question, please list the main reasons why you generally think you will regret, or not regret engaging in regular PA.” Five short open lines followed this statement, so that participants could express their views. This follows similar guidelines to general thought listing methodology (e.g., Petty and Cacioppo, 1986).

#### Behavior

Participants reported the frequency and duration of participation in mild, moderate, and vigorous intensity PA using the Godin Leisure Time Exercise Questionnaire (GLTEQ; Godin and Shephard, 1985; Godin et al., 1986). The GLTEQ was modified to correspond with current public health guidelines for moderate and vigorous intensity activity. The GLTEQ is a valid and reliable measure of PA and is one of the most commonly applied self-report measures of PA (Jacobs et al., 1993).

### Analysis Plan

Following sample descriptives, the main hypotheses were evaluated using thematic coding to categorize reasons for anticipated regret. These data were coded as total counts per theme (Petty and Cacioppo, 1986), similar to prior research (Rhodes and Blanchard, 2007). Specifically, a judge was trained to code the thoughts, identify the valence of each thought (positive negative) and to identify themes. A theme was created if at least 3% (*n* = 10) of the sample made reference to it as a reason for their reports of regret, however, we anticipated possible themes of missed opportunity (outcome values), self-conscious guilt, and external pressures. After primary coding, a second coder independently categorized reasons as well as identified the valence of each reason. The inter-rater reliability for the categories (α = 0.89) and valence (α = 1.00) were acceptable. Finally, responses were sub-coded by whether participants reported meeting Canadian PA guidelines (Tremblay et al., 2011) in order to describe the proportions of respondents by theme. To provide some falsification to this examination, we considered failure to align with a theme as outright rejection of a hypothesis, while <50% endorsement of a theme as only moderate evidence for the hypothesis. We subsequently considered differences between active and inactive participants higher than 10% as minimally meaningful (Cohen, 1992) for the exploratory analyses.

## Results

### Sample Descriptives

Participants were 120 university students (*M*_age_ = 20.64 ± 2.88; 55% female) with 2.35 ± 1.80 years of university education. Fifty-seven percent of participants were accumulating enough moderate to vigorous PA to meet the Canadian PA guidelines. Nearly the entire sample (*n* = 114; 95%) of participants said they would feel regret if they were not physically active over the next 2 weeks. Overall, participants provided a mixed range of one (*n* = 13; 11%), two (*n* = 28; 25%), three (*n* = 28; 25%), four (*n* = 14; 12%), or five (*n* = 31; 27%) reasons for regret.

### Reasons for Feeling Regret

Thematic analysis of the reasons why respondents would feel regret are provided in **Table 1**. In total, participants cited 378 reasons. Of these, 357 reasons fit into themes; the remaining 21 reasons were not classified because they were too diverse to theme (<10 reasons with similar content). Five themes emerged from the thought listing procedure, although these were also grouped as three dominant over-arching themes.

**Table 1:** Reasons for anticipated regret of physical activity (PA; *N* = 357; Active = 202, Inactive = 155).

Themes	*n*	%	Examples
Missed opportunity	247	69	

Positive expectations	138	39	“I feel better after training”
			“I know it will improve my mood after”
			“I want to be fit”
Active	88	25	“Because I want to look good”
			“I enjoy walking and running”
Inactive	50	14	“It is good for my health”
Negative expectations	109	31	“Gaining weight”
			“I would lose my gains; Lose strength”
			“I feel unhealthy physically”
Active	56	16	“I would have a harder time falling asleep”
			“I would feel bored”
Inactive	53	15	“Missed opportunity to improve my health”

Personal shame	99	28	

Evaluative	89	24	“I would be disappointed in myself”
			“I would feel lazy”
Active	44	12	“I would feel guilty”
			“I feel like I am failing myself”
Inactive	45	13	“I would feel like I let myself down”
			“Disappointing I can’t even stick to my goals”
Descriptive	10	3	“I’m an athlete”
			“I am an active person”
Active	9	2	“I am a runner”
Inactive	1	0	

External pressures	11	3	

Others	11	3	“My trainer will not be happy”
			“My tennis partner would kill me”
Active	9	2	“My team depends on my athletic capabilities”
			“I have a responsibility to exercise partner”
Inactive	2	1	“Will let down my team”


*Inactive participants defined as <150 weekly minutes of moderate + strenuous intensity PA.*

#### Missed Opportunity

The most frequent over-arching theme was an expression of the missed opportunities to achieve the benefits of regular PA (*n* = 255; 69%). In this theme, participants did not express a sense of personal regret or self-conscious shame, but did express concern for missing what they perceive the behavior bestows on them. The most frequent responses included positive expectations (*n* = 138; 39%). This was endorsed by 44% of those meeting guidelines and 32% of those not meeting guidelines. Responses were most commonly linked to the affect domain such as “I feel better after training,” or “I enjoy walking and running” but many positive expectations also included distal outcomes of PA such as “it is good for my health” or “I want to lose weight.” Negative expectations in the form of a missed opportunity were the second highest reported reasons for regret (*n* = 109, 31%). This was endorsed by 28% of those meeting guidelines and 34% of those not meeting guidelines. These were generally a re-framing of the positive expectations such as “I would lose the gains I have made,” “I would be bored,” “I would miss the chance to be healthy,” or “I would have a harder time falling asleep.”

#### Personal Shame

The next dominant theme for feeling regret reflected responses that linked to the individual’s perceived role in the performance of PA. This included 99 responses (28%) across two sub-themes. The most common sub-theme was an expression of personal shame and disappointment (*n* = 89; 25%). This was endorsed by 22% of those meeting guidelines and 29% of those not meeting guidelines. This theme included responses such as “I would be disappointed in myself,” “I would feel like I was failing myself,” “it sucks to set goals and then not keep them,” and “I would feel like I let myself down.” The second sub-theme, considerably smaller in expression (*n* = 10; 3%), was more descriptive of the personal role one has with regular PA as an identity. This was endorsed by 4% of those meeting guidelines and 1% of those not meeting guidelines. Responses included “I’m an athlete,” “I am an active person,” or “I am a runner.”

#### External Pressures

The final theme that emerged featured reasons surrounding external pressures for PA. These reasons included no expression of missed opportunity or personal concern but a sense of regret because that others would be letdown by inaction (*n* = 10; 3%). This was endorsed by 4% of those meeting guidelines and 1% of those not meeting guidelines. Examples for this theme included “My trainer will not be happy,” “My team depends on my athletic capabilities,” and “My exercise partner would shred me.”

## Discussion

Anticipated regret is a reliable predictor of future intentions and behavior (Sandberg and Conner, 2008; Conner et al., 2015), but the sources of regret are poorly understood. Thus, the purpose of this study was to theme reasons for why people responded to regret over missing regular PA. Based on the rationale and theorizing from Conner et al. (2015), we hypothesized that most people would anticipate regretting PA due to self-conscious reasons or personal standards. This hypothesis had some support. Almost a third of the reasons given for regret were aligned with personal self-conscious reasons. These were most often expressed in evaluative (e.g., I will feel like I am letting myself down) terms, thus supporting theorizing that negative emotions are anticipated pre-emptively and serve as motivation to avoid the outcome (Simonson, 1992).

A small number of reasons were also framed in terms of an identity in PA (e.g., I am an athlete). From a conceptual standpoint, future research should examine the interplay between PA identity and anticipated regret, as they may be linked via these negative emotions (Stets and Burke, 2000; Price Tangney, 2003). Manipulations where participants are asked to consider not exercising for several weeks have shown that those with exercise identities experience heightened negative affect compared to those who identify less with exercise (Strachan and Brawley, 2008; Strachan et al., 2009) and this includes personal self-conscious emotions (Flora et al., 2012). The manipulations are very similar to the phrasing of anticipated regret questions, so it seems reasonable to assume that identity may be a partial source of these anticipated affective reactions. As PA identity is also a reliable predictor of intention and behavior (Rhodes et al., 2016) an examination of these constructs may be helpful in developing theoretical depth. Interestingly, the only study to evaluate both concepts in the same model showed that anticipated regret was not related to intention independent of identity, although they shared only a modest relationship with each other (Jackson et al., 2003). Clearly more research is needed to make any judgments on how these constructs inter-relate.

A rather surprising finding from our results was that the dominant theme for anticipated regret was about the missed opportunities that regular PA bestows. These reasons were not framed in terms of any self-conscious emotions but merely that participants would miss out on an enjoyable experience, a chance to get outdoors, the potential to keep healthy, or to maintain/improve one’s figure. While, we did hypothesize that some regret would be from this missed opportunity, we did not expect such an overwhelming 69% of responses to reflect this aspect.

These results are important because they shed light on how people are perceiving regret and where this may fit in terms of our current theories. Much of regret may be overlapping with the conceptual aspects of attitudes/behavioral beliefs (Fishbein et al., 2001). Interestingly, this was also the only area where there was a marked difference in our exploratory analyses of these themes separated by past activity status (i.e., meeting PA guidelines). Active participants reported even more positive expectations than inactive participants, which is likely a consequence of stronger overall behavioral beliefs toward PA (McEachan et al., 2011). It may be that anticipated regret questions are helpful measures of these concepts because they underlie the personal value component. That is, when people are asked to answer whether PA is important or beneficial in traditional attitude questions, they may be answering in a more colloquial sense than when asked if they would regret not reaping these benefits. Typically, asking about the expectancy and value of PA in a straightforward approach has not yielded much predictive benefit above mere expectancies (Gagne and Godin, 2000; Rhodes et al., 2009a), yet an expression of this using regret, which may personalize the response, could be useful. Anticipated regret for PA does tend to correlate with attitude in the medium to large range (Conner and Abraham, 2001; Abraham and Sheeran, 2003, 2004; Jackson et al., 2003; Sheeran and Abraham, 2003; Sandberg and Conner, 2011; Wang, 2011), underscoring its shared variance. Furthermore, experimental manipulations of anticipated regret are centered upon the attitudinal domain (either through exposure to attitude questions or educational material; Abraham and Sheeran, 2004; Sandberg and Conner, 2011) and so this follows a logical sequence that anticipated regret may better reflect the personalized value of PA than standard attitude questions. Regardless, the divisive reasons for regret, between self-conscious emotions and missed opportunities, suggest that more clarity is needed in future anticipated regret measurement. We recommend that these reasons (e.g., I would regret not engaging in PA because I would feel I am letting myself down; I would regret not engaging in PA because I will have missed a chance to improve my health) be included in future assessments to help tease out the different sources of regret.

Finally, a small number of reasons (3%) for regret reflected less personal shame or missed opportunity than external social pressures. These included letting key others down (e.g., teammates, workout partner) who seemed tied to the respondent’s social network. The relatively low number of responses for these external pressures suggests this is not a frequent source of regret and would not explain much of its strong predictive value in intention and behavior. This is commensurate with subjective norm in the theory of planned behavior and introjected regulation in self-determination theory as neither construct is a reliable predictor of PA (McEachan et al., 2011; Teixeira et al., 2012). Still, the above noted recommendation to clarify the reasons for regret during assessment would help parse out external social obligations from the more frequent sources of regret in future research.

Despite the interesting and novel findings of the study, there are limitations that warrant mention. First, the sample herein is comprised of undergraduate students so these findings may not generalize to older participants. Second, our single item measure of anticipated regret may not have yielded as much from the thought-listing procedure as a multi-item measure, so replication with the more typical two- and three-items measures will help for future research on this matter. Third, the procedures in this study ask participants to consider hypothetical regret which may be different from the actual feelings of experienced regret. While hypothetical regret would still seemingly serve as the motivation for pre-emptive behavioral enactment, it would be interesting to also examine whether actual regret mirrors the expectations of participants. Fourth, the thought-listing procedure was effective at prompting a host of reasons for regret, but other procedures such as a think-aloud protocol (Fonteyn et al., 1993) may help triangulate these results.

## Author Contributions

RR conceived of the topic, wrote the paper, and assisted in the analyses. CM collected these data and engaged in the primary analyses as well as assisting in the writing.

## Conflict of Interest Statement

The authors declare that the research was conducted in the absence of any commercial or financial relationships that could be construed as a potential conflict of interest.

## Supplementary Material

**Data sheet 1 | Reasons for anticipated regret.**
